# Myocardial Energy Response to Glyceryl Trinitrate: Physiology Revisited

**DOI:** 10.3389/fphys.2021.790525

**Published:** 2021-12-31

**Authors:** William D. Watson, Peregrine G. Green, Ladislav Valkovič, Neil Herring, Stefan Neubauer, Oliver J. Rider

**Affiliations:** ^1^Oxford Centre for Magnetic Resonance Research, University of Oxford, Oxford, United Kingdom; ^2^Department for Physiology, Anatomy and Genetics, University of Oxford, Oxford, United Kingdom; ^3^Department of Imaging Methods, Institute of Measurement Science, Slovak Academy of Sciences, Bratislava, Slovakia

**Keywords:** GTN, cardiac energetics, 31P magnetic resonance spectroscopy, preload, cardiomyopathy

## Abstract

**Objective:** Although intravenous nitrates are commonly used in clinical medicine, they have been shown to increase myocardial oxygen consumption and inhibit complex IV of the electron transport chain. As such we sought to measure whether myocardial energetics were impaired during glyceryl trinitrate (GTN) infusion.

**Methods:** 10 healthy volunteers underwent cardiac magnetic resonance imaging to assess cardiac function and ^31^phosphorus magnetic resonance spectroscopy to measure Phosphocreatine/ATP (PCr/ATP) ratio and creatine kinase forward rate constant (CK *k_f_*) before and during an intravenous infusion of GTN.

**Results:** During GTN infusion, mean arterial pressure (78 ± 7 vs. 65 ± 6 mmHg, *p* < 0.001), left ventricular (LV) stroke work (7,708 ± 2,782 vs. 6,071 ± 2,660 ml mmHg, *p* < 0.001), and rate pressure product (7,214 ± 1,051 vs. 6,929 ± 976 mmHg bpm, *p* = 0.06) all fell. LV ejection fraction increased (61 ± 3 vs. 66 ± 4%, *p* < 0.001), with cardiac output remaining constant (6.2 ± 1.5 vs. 6.5 ± 1.4 l/min, *p* = 0.37). Myocardial PCr/ATP fell during GTN infusion (2.17 ± 0.2 vs. 1.99 ± 0.22, *p* = 0.03) with an increase in both CK *k_f_* (0.16 ± 0.07 vs. 0.25 ± 0.1 s^−1^, *p* = 0.006) and CK flux (1.8 ± 0.8 vs. 2.6 ± 1.1 μmol/g/s, *p* = 0.03).

**Conclusion:** During GTN infusion, despite reduced LV stroke work and maintained cardiac output, there was a 44% increase in myocardial ATP delivery through CK. As PCr/ATP fell, this increase in ATP demand coincided with GTN-induced impairment of mitochondrial oxidative phosphorylation. Overall, this suggests that while GTN reduces cardiac work, it does so at the expense of increasing ATP demand beyond the capacity to increase ATP production.

## Introduction

The heart has an extremely high turnover of energy, requiring around 6 kg of adenosine triphosphate (ATP) per day in order to perform contraction and relaxation. As ATP production is reduced in heart failure (Neubauer, 2007), many current treatments are aimed at lowering myocardial energy use by decreasing cardiac work.

Glyceryl trinitrate (GTN) is one such drug that has been used for nearly 150 years to treat cardiovascular disease. Nitrates predominantly dilate systemic veins, increasing the capacitance of these vessels and reducing venous return to the heart, thereby lowering ventricular filling and preload. GTN achieves this *via* activation of cGMP-dependent protein kinase, without donating significant amounts of the free radical nitric oxide (Kleschyov et al., 2003). At higher doses, they have effects on arterial tone, lowering systemic vascular resistance, and therefore afterload. In acute heart failure, this is therapeutic, with the reduction in preload reducing pulmonary venous congestion and the reduction in afterload reducing cardiac work.

However, despite these beneficial hemodynamic effects in fluid overloaded heart failure, studies in healthy, euvolemic humans have showed that intravenous nitrate increased myocardial oxygen usage despite cardiac work being the same, or reduced (Brachfeld et al., 1959). This implies more ATP is being used to sustain the same or less cardiac work and as such that cardiac efficiency is lower. In addition to this, it is reported in animal models that GTN causes nitric oxide-induced inhibition of complex IV of the electron transport chain, resulting in inhibition of mitochondrial ATP production by oxidative phosphorylation (Somani et al., 1969; Brown and Cooper, 1994). When considering the two effects, GTN may result in energetic stress with reduced ATP production in the face of increased demand if the patient is initially euvolemic and the GTN causes a reduction in preload which compromises myocardial stretch necessary for the optimal operation of the Frank-Starling relationship. This has not been investigated in humans to date but would provide valuable insight into the effects of this well-established medication.

As a result, we used the combination of cardiac magnetic resonance imaging (MRI) and phosphorus magnetic resonance spectroscopy to record cardiac function and energy metabolism in healthy volunteers before and during GTN infusion.

## Materials and Methods

The study was approved by the University of Oxford Medical Sciences Interdivisional Research Ethics Committee (reference R64397/RE001) and was in accordance with the Declaration of Helsinki. 10 healthy volunteers were recruited *via* word of mouth, emails to departmental mailing lists, social media postings, and posters located in University Departments. Participant characteristics are in Table 1.

**Table 1:** Participant characteristics for the study cohort.

	Mean	Range
Age	38	26–63
Height (m)	1.76	1.64–2.09
Weight (kg)	71	59–107
BMI	22.6	18.5–27.8

### Exclusion Criteria

Exclusion criteria were as follows: any history of cardiac disease (including valvular heart disease, myocardial impairment, or coronary artery disease), any history of metabolic disorder (defined as a history of diabetes mellitus, hypothyroidism, or anemia), any contra-indications to nitrate-based medications, any contra-indication to MRI scanning, or involvement in any other research projects where the procedures may affect the outcomes of the study.

### Anthropometric and Biochemical Assessment

Height and weight were measured using digital scales and a gradated analog measurement system, respectively. Non-invasive blood pressure was measured according to standardized methods (average of 3 supine measurements with an automatic sphygmomanometer, Carescape V100, GE).

### Cardiac Magnetic Resonance

All scans were performed at 3.0 Tesla (Siemens Healthineers, Erlangen, Germany), before and during GTN infusion.

### Cardiac Imaging

Cardiac imaging to quantify ventricular volumes and function was acquired using a steady-state free precession sequence (echo time, 1.5 ms; repetition time, 3 ms), which was performed with cardiac triggering and during end-expiratory breath-hold. Cardiac volumes were acquired with horizontal long axis, vertical long axis and short axis cines. Endocardial and epicardial left ventricular contours were drawn manually and analyzed using a semi-automated system (cmr42, Circle Cardiovascular Imaging Inc.) as previously described (Rayner et al., 2021). All cardiac imaging data were analyzed blinded to participant and scan details.

Left ventricular stroke work was calculated as stroke volume × mean arterial pressure, cardiac minute work was calculated as the product of stroke volume, heart rate, and systolic blood pressure, and rate pressure product was calculated as heart rate × systolic blood pressure.

### ^31^P Magnetic Resonance Spectroscopy

Phosphocreatine/ATP ratio (PCr/ATP) was measured with a ultra-short echo time three-dimensional chemical shift imaging sequence as previously described (Tyler et al., 2009). Participants lay prone over a dual-tuned ^1^H/^31^P surface coil with their left ventricle positioned over the center of the coil at the magnet iso-center with proton localisers to ensure correct positioning. Parameters included acquisition matrix size 16 × 8 × 8 voxels, field of view 240 × 240 × 200 mm (Brachfeld et al., 1959), nominal voxel size 11.25 ml, 10 averages at the center of k-space, fixed TR per subject (910–1,010 ms depending on specific absorption rate constraints), and center frequency 250 Hz from phosphocreatine (PCr). The PCr/ATP ratio reported is the blood- and saturation-corrected PCr/average ATP ratio from the basal septal voxel. Spectral analysis was performed using the OXSA toolbox which uses a MATLAB implementation of the Advanced Method for Accurate, Robust and Efficient Spectral fitting algorithm (Purvis et al., 2017).

Measurements of the CK forward rate constant k*_f_* were measured using a Triple Repetition Saturation Transfer (Schar et al., 2010) sequence with stress saturation transfer (Clarke et al., 2019) extension, as previously described (Peterzan et al., 2020; Rayner et al., 2020) and validated in human subjects (Clarke et al., 2019). A transmit/receive ^31^P MRS coil with 17 cm loop transmit coil and 11 cm loop receive coil (Pulseteq, United Kingdom) was used.

Briefly, participants were scanned supine, and localisers were used to confirm coil position; then, a 1-dimensional chemical shift imaging sequence using frequency-sweep cycled adiabatic half passage excitation pulse (Schar et al., 2010) was used to acquire 4 sets of ^31^P spectra. The first sequence had no saturation (TR 15 s, 2 averages, scan time ~ 9 min), a second and third used selective saturation of the *γ*-ATP peak (first sequence TR 1.5 s, 18 averages, scan time ~ 11 min; second sequence TR 9.5 s, 8 averages, scan time ~ 21 min) and a final “control saturation” sequence with saturation mirrored to the other side of the PCr peak (TR 15 s, 2 averages, scan time ~ 9 min). Spectral analysis was performed using an in-house software. The pseudo-first-order unidirectional rate constant (*k_f_*) of creatine kinase in the forward (ATP producing) direction was calculated according to the following:


$$k_{f}^{CK}=\frac{1}{T_{1}^{*}}\left(\frac{M_{PCr}^{Ctrl}}{M_{PCr}^{\prime }}-1\right)$$

Phosphocreatine concentration (PCr) was estimated by multiplying PCr/ATP by the literature values for (ATP) in healthy subjects [5.7 μmol/g wet weight (Weiss et al., 2005)] and forward creatine kinase flux was calculated as (PCr) × *k_f_*.

### GTN Infusion

1 mg/ml GTN infusion was started at a rate of 3 ml/Hr and titrated at 5-min intervals with a target of a 15 mmHg fall in mean arterial pressure (but not to lower it below 70 mmHg) and then maintained during scanning.

### Statistical Analysis

Statistical analysis was performed using GraphPad Prism (GraphPad software, United States). All data were subjected to Kolmogorov-Smirnov tests to establish normal distribution and are presented as mean ± standard deviation. Paired-equal variance Student’s *t*-tests were performed for pre and during infusion data sets. Correlations were assessed with Pearson’s correlation analysis. A probability of *p* < 0.05 was considered significant.

## Results

### Hemodynamics and Ventricular Volumes

**Figure 1:**
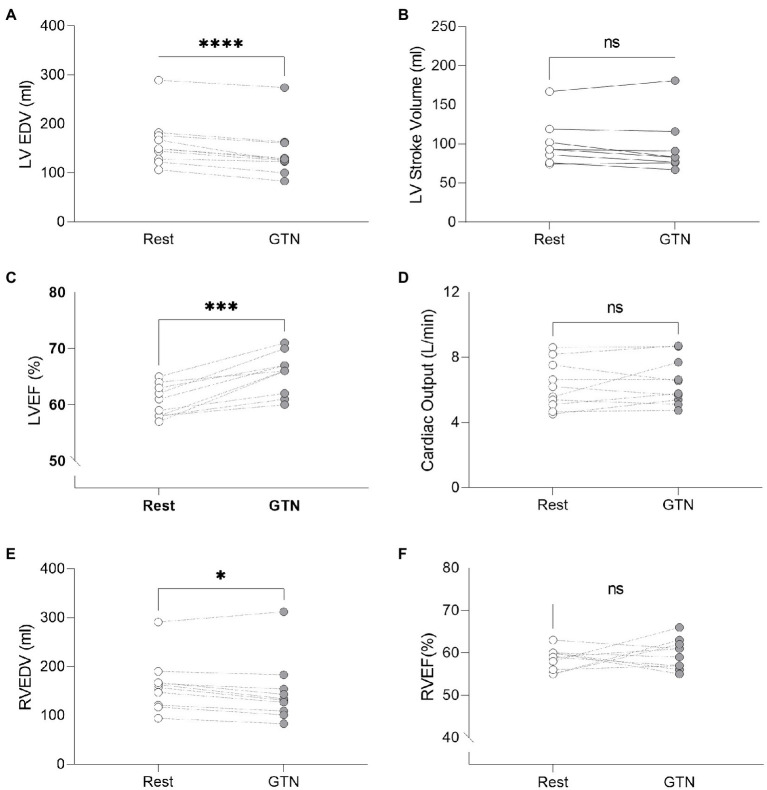
Left and Right ventricular measurements at rest and during GTN infusion. **(A)** Left ventricular end diastolic volume, **(B)** Left ventricular stroke volume, **(C)** Left ventricular ejection fraction, **(D)** Cardiac output, **(E)** Right ventricular end diastolic volume, and **(F)** Right ventricular ejection fraction. ^*^p < 0.05, ^***^p < 0.001, and ^****^p < 0.0001.

GTN infusion caused a reduction in left (by 20 ml, *p* < 0.001) and right (by 13 mls, *p* = 0.01) ventricular end-diastolic volume and right atrial area (by 3 cm^2^ *p* = 0.04, Table 2; Figure 1). While cardiac pressures were not measured directly, these reductions in chamber size were in line with GTN-induced vasodilatation and preload reduction. Mean arterial pressure fell by an average of 13 mmHg (*p* < 0.05), in line with its additional known effects on afterload.

**Table 2:** Left and right heart measurements at rest and during GTN infusion.

	Baseline	During GTN	Value of *p*
Left ventricular end-diastolic volume (ml)	161 ± 51	141 ± 52	**<0.001**
Left ventricular end-systolic volume (ml)	64 ± 22	48 ± 18	**<0.001**
Left ventricular stroke volume (ml)	97 ± 30	93 ± 35	0.14
Left ventricular ejection fraction (%)	61 ± 3	66 ± 4	**<0.001**
Cardiac output (L/min)	6.24 ± 1.46	6.49 ± 1.43	0.37
Cardiac index (L/min m^2^)	3.35 ± 0.5	3.47 ± 0.3	0.4
Right atrial area (cm^2^)	23.3 ± 7.8	20.9 ± 6.7	**0.04**
Right ventricular end diastolic volume	161 ± 54	148 ± 64	**0.01**
Right ventricular end systolic volume	70 ± 26	61 ± 29	**0.03**
Right ventricular ejection fraction	59 ± 3	60 ± 3	0.46

Significant differences on paired *t*-test are highlighted.

In contrast, both heart rate (by 9 beats per minutes) and left ventricular ejection fraction (by 5%, both *p* < 0.001) were increased during GTN. This resulted in maintenance of cardiac output and cardiac index which were unchanged (*p* = 0.37 and 0.40, respectively).

**Figure 2:**
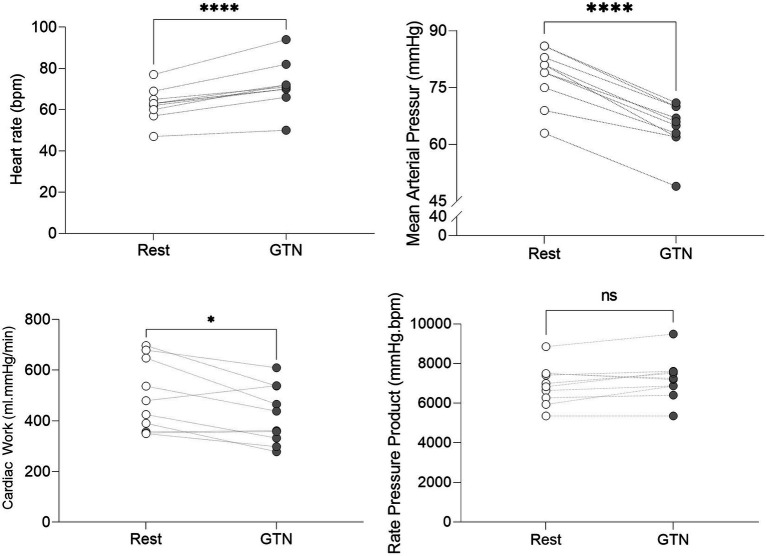
Hemodynamic values at rest and during GTN infusion (values averaged over 5 min). ^*^p < 0.05 and ^****^p < 0.0001.

Despite this maintenance of cardiac output, calculated left ventricular stroke work (by 21%, p < 0.001) and cardiac work (by 14%, *p* = 0.02) both fell, driven primarily by the lower mean arterial pressure (Table 3; Figure 2).

**Table 3:** Hemodynamic values at rest and during GTN infusion (values averaged over 5 min).

	Baseline	During GTN	Value of *p*
Mean arterial pressure (mmHg)	78 ± 7	65 ± 6	**<0.0001**
Heart rate (bpm)	63 ± 7	72 ± 11	**<0.0001**
Rate pressure product (mmHg bpm)	6,929 ± 976	7,214 ± 1,051	0.06
LV Stroke work (ml mmHg)	7,708 ± 2,782	6,071 ± 2,660	**<0.0001**
Cardiac minute work (L mmHg bpm)	491 ± 140	422 ± 114	**0.02**

Significant differences on paired *t*-test are highlighted.

Overall, this is in keeping with the known effects of GTN and shows that while heart rate and left ventricular ejection fraction rose to maintain cardiac output, mechanical or external cardiac work was lower during GTN.

### ^31^P Magnetic Resonance Spectroscopy

Two out of the 10 participants spectra for creatine kinase rate constant calculations had values of >1 for standard deviation of uncertainty of fit and were hence discarded.

**Figure 3:**
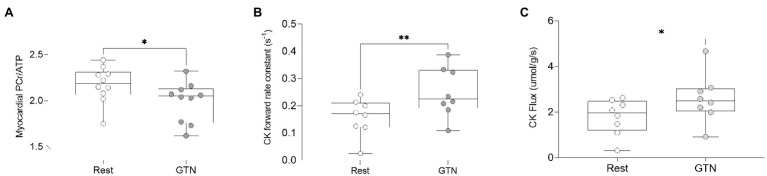
The effects of GTN infusion on Myocardial energetics as assessed by ^31^P magnetic resonance spectroscopy showing **(A)** myocardial Phosphocreatine/ATP (PCr/ATP) ratio, **(B)** creatine kinase pseudo-first-order forward rate constant, and **(C)** creatine kinase flux. ^*^p < 0.05 and ^**^p < 0.01.

During GTN infusion, there was a reduction in the PCr/ATP ratio (by 8%, *p* = 0.03, Table 4; Figure 3). In addition, there were increases in both CK pseudo-first-order rate constant (by 58%, *p* < 0.01) and CK flux (by 45%, *p* = 0.03, Figure 3) during GTN infusion.

**Table 4:** ^31^P magnetic resonance spectroscopy measurements of myocardial energetics at rest and during GTN infusion.

	Baseline	During GTN	Value of *p*
PCr/ATP ratio	2.17 ± 0.2	1.99 ± 0.22	**0.03**
CK first order rate constant s^−1^	0.158 ± 0.068	0.249 ± 0.091	**0.006**
CK flux μmol/g/s^−1^	1.79 ± 0.79	2.59 ± 1.07	**0.03**

Significant differences on paired t-test are highlighted.

**Figure 4:**
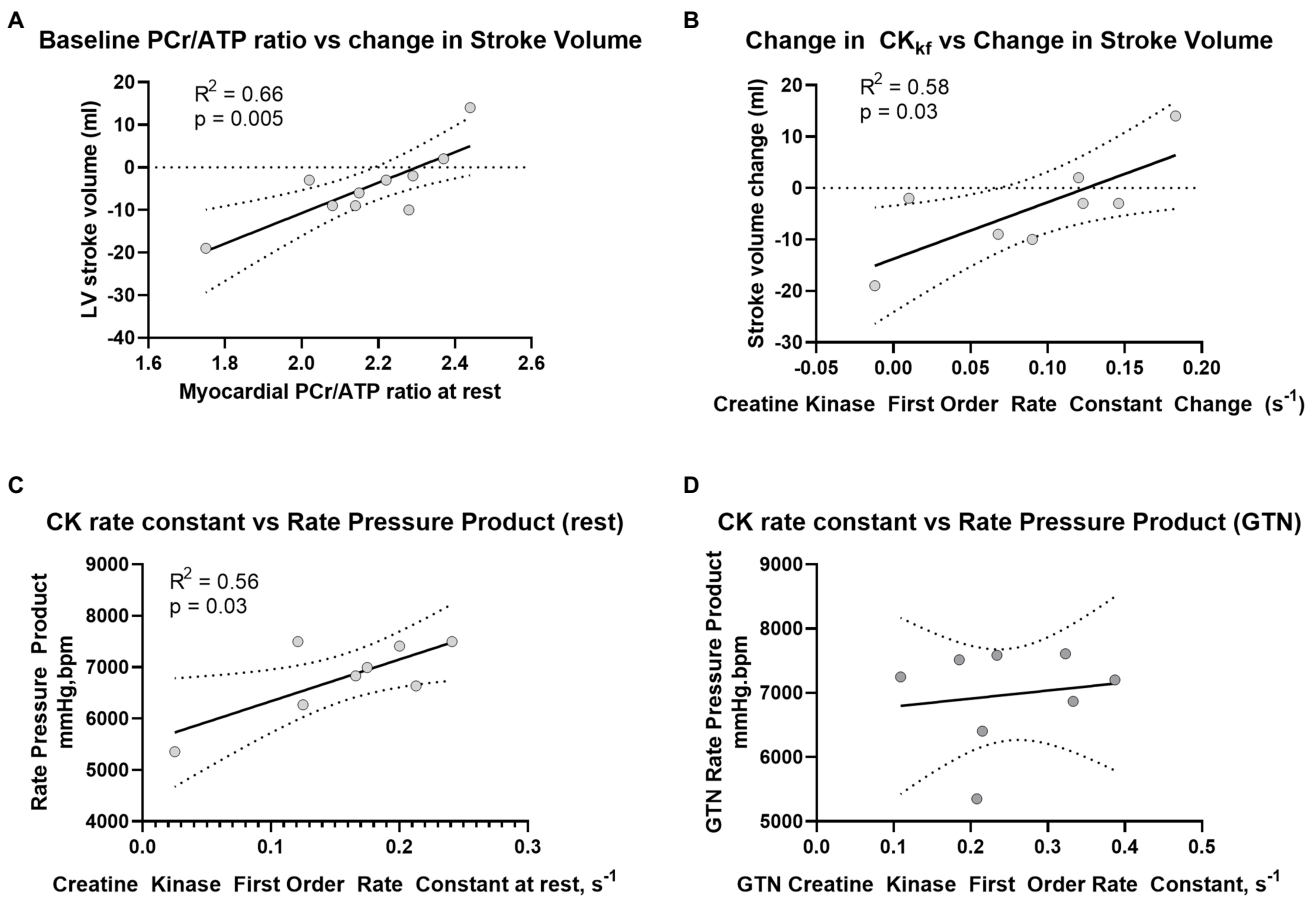
Relationship between **(A)** PCr/ATP and LV Stroke volume at rest, **(B)** change in CK rate constant and change in LV stroke volume during GTN, **(C)** Rate Pressure Product at rest and CK rate constant before, and **(D)** during GTN infusion.

Prior to the administration of GTN, and as expected, there was a positive correlation between CK rate constant and rate pressure product (*R*^2^ = 0.56, *p* = 0.03, Figure 4A), as well as a trend toward a positive correlation between CK flux and rate pressure product (*R*^2^ = 0.4, *p* = 0.09). There was also a strong positive correlation between the resting PCr/ATP value and the change in stroke volume during GTN (*R*^2^ = 0.66, *p* = 0.004, Figure 4B), where lower resting PCr/ATP levels were positively correlated with the largest drop in stroke volume, with higher resting PCr/ATP being associated with increased stroke volume during GTN. This relationship was similar to that seen with the change in CK forward rate constant during GTN, (*R*^2^ = 0.578, *p* = 0.028, Figure 4C), where again the smallest increase was related to the largest falls in stroke volume and the largest increases to increased stroke volume.

Interestingly, the observed relationship between CK activity and rate pressure product seen at rest was lost during GTN infusion (Figure 4D).

Overall, this suggests that myocardial delivery through CK is higher during GTN infusion and that as phosphocreatine levels fall, this increase is greater than the rate of ATP production from mitochondrial oxidative phosphorylation. In addition, it suggests that the tight coupling of CK activity to work, seen at rest, is lost during GTN infusion.

## Discussion

GTN is one of the most commonly used cardiovascular drugs. In this study, we have used *in vivo* ^31^P magnetic resonance spectroscopy to assess human ATP kinetics during GTN infusion. We have shown that despite its hemodynamic effect of reducing mean arterial pressure and cardiac work, ATP delivery through CK is increased; however, GTN simultaneously inhibits the rate of ATP production from mitochondrial oxidative phosphorylation.

### Effects of GTN Infusions on Cardiac Function and Hemodynamics

In this study, GTN infusion resulted in a reduced mean arterial pressure and increased heart rate and left ventricular ejection fraction. The fall in left and right ventricular end-diastolic volume and right atrial size are likely to reflect a fall in preload caused by the venoparesis. The observed increased in contractility (here seen as increased left ventricular ejection fraction) and heart rate reflect the known baroreceptor reflex response to reduced preload and reduced aortic stretch during GTN that compensates for the fall in preload and afterload to preserve cardiac output (which was unchanged in this experiment) and maintain tissue perfusion. Overall, however, despite this unchanged cardiac output, left ventricular stroke work was reduced.

### The Effects of GTN Infusion on Myocardial High-Energy Phosphate Metabolism

#### Creatine Kinase Flux

Although it might be expected that as cardiac output was maintained, and many calculated indices of cardiac work (rate pressure product, stroke work, and cardiac work) fell or remained constant during GTN, that ATP delivery would also remain similar or reduced, this was not the case.

Despite maintained cardiac output, and reduced stroke work during GTN, there was a significant increase in both the creatine kinase forward rate constant and creatine kinase flux. This would suggest that ATP delivery through creatine kinase was increased significantly. While this is unexpected as ATP usage and cardiac work are tightly coupled, it does however provide an explanation for the previous human studies showing global myocardial oxygen use is increased during GTN infusion (Brachfeld et al., 1959; indeed the average observed increase in myocardial oxygen consumption in this study of 51% closely matches our average observed CK flux increase of 45%). CK had been previously shown to be inhibited in rat hearts by a NO donor (Gross et al., 1996); however, GTN may not donate NO in significant quantities (Kleschyov et al., 2003), explaining the disparity here. In addition, the loss of the relationship between CK activity and rate pressure product during GTN infusion would suggest a decoupling of ATP delivery and cardiac work. The mechanism behind this observation remains elusive.

#### Myocardial ATP Production

During GTN infusion, we have showed that the myocardial PCr/ATP ratio is reduced. This is a surprising finding, as the PCr/ATP in the normal heart is reported almost universally to be maintained during all but extreme inotropic stress (Pluim et al., 1998; Rayner et al., 2021). This suggests during GTN infusion that oxidative phosphorylation is being impaired and that ATP use is greater than the rate of ATP production, with phosphocreatine pool depletion occurring to maintain ATP levels.

One explanation may lie in the fact that GTN has been shown to cause nitric oxide-induced inhibition of complex IV of the respiratory chain (Brown and Cooper, 1994) and with it impaired oxidative phosphorylation in isolated mitochondria (Boime and Edmund, 1971). This also explains the reduction in oxygen uptake seen in isolated myocytes (Somani et al., 1969; Gross and Hardman, 1975).

By using ^31^P magnetic resonance spectroscopy to measure both ATP delivery and PCr/ATP, this study can also reconcile the apparently contradictory findings that in the intact human heart MVO_2_ is elevated, but in the isolated cell it is reduced. Although total ATP demand, and as such oxygen demand is increased by GTN (in this study seen as increased CK flux), mitochondrial oxidative phosphorylation is impaired by GTN (seen as a failure to maintain PCr/ATP levels).

## Limitations

In this study, rate pressure product and other non-invasive derived measures have been used as a surrogate (Nelson et al., 1974) for myocardial work and oxygen consumption. However, this discounts work done by the right ventricle, substitutes pressure for wall tension stress, and discounts “internal” cardiac work used to facilitate diastole and essential cellular ATPases.

While the creatine kinase shuttle is thought to be the main transfer mechanism involved in transferring ATP from the mitochondrion to the myofibril, using the rapid diffusion capability of phosphocreatine to transfer a phosphate group into ATP at the myofibril, there are several other mechanisms that can shuttle ATP, including simple diffusion, which are not measured here. However, as they only present ~15% of ATP transfer (Peterzan et al., 2017) we feel this remains reflective of the major biological pathways.

While we have shown changes in high-energy phosphate metabolism here, the study was not designed to look at changes in myocardial substrate metabolism, for instance anaerobic glycolysis. Future studies measuring myocardial metabolism with hyperpolarized pyruvate or PET tracers, or measuring myocardial pH may give further information here.

## Conclusion

This study is the first study to use ^31^P magnetic resonance spectroscopy to assess ATP kinetics during GTN infusion in humans. We have shown that despite its hemodynamic effect to reduce mean arterial pressure and cardiac work, ATP delivery through CK is increased by GTN, while simultaneously the capacity to increase rate of ATP production from mitochondrial oxidative phosphorylation in response to increased demand is reduced by GTN. Using ^31^P in this way has allowed a greater understanding of the actions of this commonly prescribed medication.

## Data Availability Statement

The raw data supporting the conclusions of this article will be made available by the authors, without undue reservation.

## Ethics Statement

The studies involving human participants were reviewed and approved by University of Oxford Medical Sciences Interdivisional Research Ethics Committee (reference R64397/RE001). The patients/participants provided their written informed consent to participate in this study.

## Author Contributions

WW conceived the research and developed it with help from the other authors. WW and PG performed the experiments. LV developed the MRS sequences and assisted in analysis. OR, NH, and SN aided in drafting the manuscript. All authors contributed to the article and approved the submitted version.

## Funding

WW and PG are funded by the BHF Clinical Research Training Fellowships (FS/17/48/32907 and FS/18/50/33807). NH is a BHF Senior Clinical Research Fellow (FS/SCRF/20/32005). OR is funded by BHF intermediate research fellowship (FS/16/70/32157). LV is funded by a Sir Henry Dale Fellowship from the Royal Society and The Wellcome Trust (221805/Z/20/Z).

## Conflict of Interest

The authors declare that the research was conducted in the absence of any commercial or financial relationships that could be construed as a potential conflict of interest.

## Publisher’s Note

All claims expressed in this article are solely those of the authors and do not necessarily represent those of their affiliated organizations, or those of the publisher, the editors and the reviewers. Any product that may be evaluated in this article, or claim that may be made by its manufacturer, is not guaranteed or endorsed by the publisher.
